# Long‐Term Outcomes of Invasive vs Noninvasive Treatment for Intermittent Claudication: A Systematic Review and Meta‐Analysis

**DOI:** 10.1002/cesm.70053

**Published:** 2025-10-03

**Authors:** Anas Elmahi, Nathalie Doolan, Mohiedin Hezima, Anwar Gowey, Daragh Moneley, Seamus McHugh, Sayed Aly, Peter Naughton, Elrasheid A. H. Kheirelseid

**Affiliations:** ^1^ Department of Vascular Surgery Beaumont Hospital and RCSI Dublin Ireland

**Keywords:** intermittent claudication, invasive treatment, meta‐analysis, noninvasive treatment, randomized controlled trials, systematic review

## Abstract

**Background:**

Intermittent claudication (IC) is a hallmark symptom of peripheral arterial disease (PAD), causing pain and discomfort during physical activity caused by reduced blood flow to the lower extremities. The condition significantly impairs mobility and quality of life (QoL) in affected individuals. Treatment options for IC range from conservative approaches, including best medical therapy (BMT) and supervised exercise therapy (SET), to invasive interventions like angioplasty and open re‐vascularization.

**Aim:**

This meta‐analysis and systematic review seek to assess the long‐term results of invasive procedures concerning Noninvasive treatments for the management of patients with IC.

**Methods:**

A comprehensive search was conducted in October 2024 across databases containing PubMed, MEDLINE, Cochrane Library, Embase, and Scopus. Randomized controlled trials (RCTs) comparing invasive interventions to Noninvasive treatments were included. Primary outcomes were quality of life (QoL), ankle‐brachial pressure index (ABPI), and maximum walking distance (MWD). Secondary outcomes were major adverse cardiovascular events (MACE), mortality, complications, and re‐intervention rates. Data analysis was conducted using the Cochrane Review Manager 5. Follow‐up duration was between 2 and 7 years, longest available between 2 and 7 years; prioritized 2 years when present.

**Results:**

A total of 11 RCTs with 1379 patients were included in the analysis. Invasive treatments demonstrated a significant improvement in MWD and ABPI compared to Noninvasive treatments (MWD pooled Mean Difference (MD) = 64.94 [10.77, 115.12] 95% CI, *p* = .02, 5 studies, and ABPI pooled MD = 0.15 [0.04, 0.26] 95% CI, *p* = .006, 5 studies). However, invasive interventions were associated with a higher rate of complications, including increased amputation risk (Pooled odds ratio (OR) = 2.46 [0.44, 13.94] 95% CI, *p* = .31, 3 studies), though this was not statistically significant. Long‐term rates were higher in the Noninvasive treatment group (Pooled OR: 0.56 [0.33, 0.97] 95% CI, *p* = .04).

**Conclusions:**

Both invasive and Noninvasive treatments are effective in managing IC. Invasive treatments provide greater improvement in blood flow and walking distance, but the risk of complications and re‐interventions should be considered in treatment decisions. Further research with larger sample sizes and designed for long‐term assessment is needed to assess the cost‐effectiveness and long‐term outcomes of invasive treatments.

## Summary

Intermittent claudication (IC) is a painful condition caused by poor blood flow to the legs, making it hard for people to walk. It commonly affects older adults and those with peripheral artery disease (PAD). PAD is a common condition where a build‐up of fatty deposits in the arteries restricts blood supply to leg muscles. There are two main ways to treat IC:

1. Noninvasive treatments like medications and supervised exercise.
2. Invasive treatments like angioplasty or surgery to open blocked arteries.

This study looked at 11 clinical trials involving 1379 patients to compare the long‐term results of these treatments. We found that invasive treatments helped patients walk farther and improved blood flow in their legs more than Noninvasive treatments. However, invasive treatments also came with higher risks, including complications and the possible need for further procedures. On the other hand, Noninvasive treatments were safer but often did not provide as much improvement. Some patients who started with Noninvasive treatments eventually needed surgery later. In conclusion, both treatments have their pros and cons. The best choice depends on each patient's health condition, preferences, and long‐term needs. Doctors and patients should work together to choose the most suitable option.

## Graphical abstract

In patients with leg pain from blocked arteries (intermittent claudication), this study shows that surgical procedures improve walking ability and blood flow more than exercise or medication alone but carry higher risks. The findings help guide doctors and patients in choosing the best treatment based on individual needs and risks.

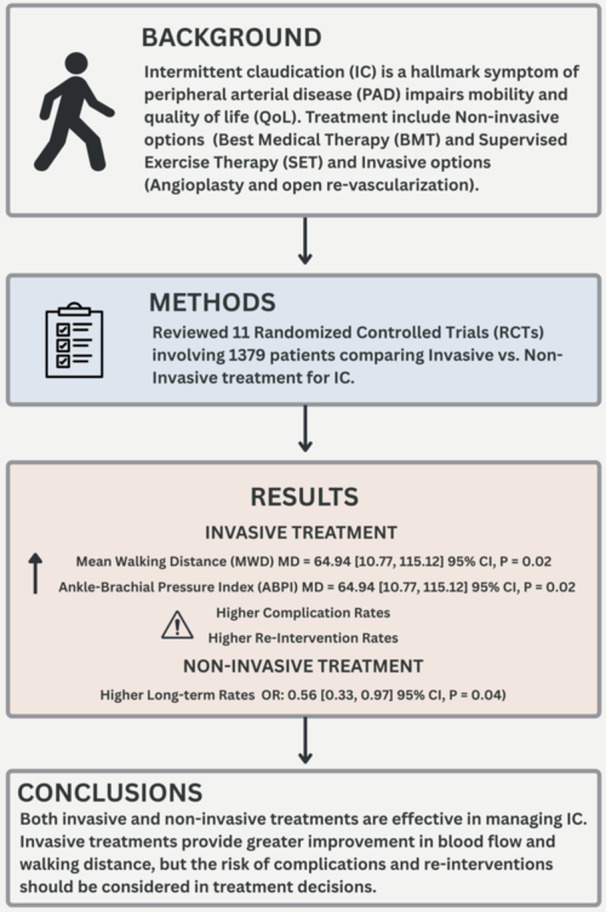

## 1 Introduction

### 1.1 Epidemiology of Intermittent Claudication

The most characteristic symptom of peripheral arterial disease (PAD) is the presence of intermittent claudication (IC) during physical activity, suffering in the lower extremities caused by the insufficiency of blood circulation. In the Western world, PAD is prevalent in 4.3% of the population aged over 40 years, and even higher in people over 70 years of age (14.5%) [1]. IC has significant impacts in terms of impaired mobility and quality of life, and it restricts the capability of patients to conduct their daily activities. Furthermore, IC increases the risk of developing critical limb ischemia in 5 years, which is clinically relevant [2]. Considering that IC is a significant burden, it is necessary to implement effective management measures to positively affect patient outcomes and impede disease progression.

### 1.2 Current Treatments

IC can be treated through Noninvasive and invasive methods. Noninvasive treatment includes best medical therapy (BMT) (pharmacotherapy: antiplatelets, statins, and risk factor reduction) and supervised exercise therapy (SET) that leads to exercise capacity and cardiovascular risk decrease [1, 3, 4, 5, 6]. Relatively, SET is more effective when compared with unsupervised exercise, but its effectiveness is closely related to the nature and format of programs, and their duration and intensity [7, 8]. The role of invasive therapy, including percutaneous transluminal angioplasty, stenting, or open revascularization, is to achieve the patency of arteries and improvement of the perfusion of legs [7, 8]. Such interventions have been known to have immediate effects of restoring walking performance and ankle‐brachial pressure index (ABPI) [9, 10, 11]. They, however, have risks of developing complications, i.e., infections, restenosis, and, in extreme incidences, amputation. The treatment decision is based on the nature of the patients, the severe condition of the disease, and clinical recommendations [7, 8].

### 1.3 Gaps in Evidence

Although the roles of both Noninvasive and invasive treatments have been defined, there has been ambiguity in the effectiveness of the long‐term comparisons of the two types of treatment. Although SET and BMT have been advised as first‐line interventions, the initiative in implementing the best program (e.g., length, frequency) and patient adherence is not well known because of its limited supervised lending facilities and cost implications [7, 8]. The invasive treatments have exhibited short‐term improvement in functional status, but the long‐term insights of the method regarding the techniques of Noninvasive application are controversial, especially in the occurrence of complications [9, 10, 11]. Studies conducted before have shown mixed results, with some indicating that there remain improvements in mobility and living standards over time, whereas some indicated increased reintervention rates among conservatively managed patients [7, 10, 11]. Furthermore, the cost‐effectiveness of the invasive and Noninvasive treatments should be examined to assist in decision‐making in clinical settings.

### 1.4 Aim of the Study

This systematic review and meta‐analysis are proposed to compare the long‐term outcome of invasive (e.g., angioplasty, revascularization) with that of Noninvasive (e.g., SET, BMT) treatment in patients with IC, based on the available data in the randomized controlled trials (RCTs). The integration of the existing evidence on major outcomes, including, but not limited to, Maximum Walking Distance (MWD), ABPI, quality of life (QoL), and complication rates, is aimed at the provision of substantial evidence to clinicians to guide decisions in treatment and improve patient‐centered care of IC.

## 2 Methodology

### 2.1 Study Design

As stated in the Cochrane Handbook for Systematic Reviews of Interventions, the Preferred Reporting Items for Systematic Reviews and Meta‐Analyses (PRISMA) guidelines were followed in the conduct of this systematic review and meta‐analysis. This study was prospectively registered with the International Prospective Register of Systematic Reviews (PROSPERO; registration ID: CRD42023494962) on 18 December 2023. The protocol outlined the objectives, eligibility criteria, search strategy, and planned analyses before data extraction and synthesis. No post‐hoc analytic decisions that could introduce bias were made beyond what was specified in the preregistered protocol. Ethical or Institutional Review Board approval was not needed for this study. The major focus was to undertake a systematic review that focused on the utility of invasive interventions versus noninvasive therapies in patients with IC using RCTs only. The identification process also included the selection criteria that were meticulously determined to ensure a reduced bias level and improved productivity in the analysis of the data extracted from the included articles.

### 2.2 Search Strategy

A comprehensive search of multiple databases was conducted in October 2024. The databases, including PubMed, MEDLINE, Cochrane Library, Embase, and Scopus, were searched. The search strategy aimed to identify all relevant RCTs comparing invasive treatments (such as angioplasty and revascularization) with Noninvasive treatments (such as SET or BMT). The search was conducted without date or language restrictions to ensure a comprehensive capture of studies from inception to the present. Furthermore, a manual check of the included studies’ bibliographies was conducted to uncover additional relevant research that might have passed through the openings of the first search Table 1.

**Table 1:** Exclusion and inclusion criteria of included studies.

Inclusion criteria	Exclusion criteria
Studies included adult patients diagnosed with intermittent claudication who were treated with either invasive or conservative management.	Studies that did not directly compare invasive treatments with conservative treatments.
Studies explored the intervention group/patients who underwent invasive treatments for intermittent claudication.	Case reports, case series, observational studies, and qualitative studies.
Studies included Noninvasive treatments like a control group/patients treated conservatively with SET and/or best medical therapy (BMT).	Interventions including experimental regenerative, shockwave, and thermal therapies were excluded unless they were part of an RCT comparing them to SET or BMT in line with clinical guidelines.
Studies specified the severity or clinical stage of peripheral artery disease (e.g., Fontaine II, Rutherford 1–3 or Fontaine IIb) to confirm relevance to intermittent claudication and results generalizability.	Studies not available in English or those that did not report primary or secondary outcomes relevant to the review.
Studies explored the primary outcomes of interest, including QoL, MWD, and ABPI. Secondary outcomes included MACE, mortality, complications, and the need for re‐intervention.	
Only RCTs were included.	

### 2.3 Selection Criteria

The inclusion and exclusion criteria for studies were as follows:

The inclusion and exclusion criteria of the individual included studies were summarized in Table 2.

**Table 2:** Inclusion and exclusion criteria of the individual included studies.

Study	Inclusion criteria	Exclusion criteria
Djerf et al. [12]	Data from the IRONIC trial	Data from the IRONIC trial
Fakhry et al. [7]	Not Reported (NR)	NR
Greenhalgh [13]	No age/gender limits; Positive Edinburgh Claudication Questionnaire; ABPI < 0.9 or > 0.9 with stress test; Aortoiliac or femoropopliteal lesion amenable to PTA	Mild symptoms not needing angioplasty; Critical limb ischemia; Severe comorbidities prohibiting exercise
Gunnarsson et al. [14]	Age > 18; Stable IC > 6 months (Fontaine IIb); Walking distance < 500 m; De novo/restenotic SFA lesion; Patent popliteal and tibial runoff arteries	Stroke (last 3 months); Aneurysms; Previous stents at same site; Poor inflow; Target artery < 4 mm; CLTI; Life expectancy < 24 months; Previous enrollment
Mazari et al. [15]	Unilateral IC due to femoropopliteal lesion; Eligible for angioplasty; Able to exercise	Aortoiliac or mixed lesions; Critical limb ischemia; Severe comorbidities
Nordanstig et al. [16]	Stable lifestyle‐limiting IC > 6 months; Age ≤ 80	Need for invasive treatment; Bodyweight > 120 kg; Mild symptoms; ≥ 2 failed revascularizations; Inability to complete HRQoL forms; Need for distal revascularization
Nylaende et al. [17]	Age < 80; Symptomatic IC > 3 months; ABI < 0.9; Angioplasty‐feasible lesion; PFWD < 400 m; Able to exercise	Previous vascular surgery; Diabetic ulcers; Renal insufficiency (> 150 mmol/L creatinine); On anticoagulants; Physical/mental disorders
Perkins et al. [18]	Stable unilateral claudication; Failed conservative treatment > 3 months; Angiography‐suitable lesion; Walking distance < 375 m	Death; Lost to follow‐up
van den Houte et al. [19]	PAD diagnosis; clinical stage of peripheral artery disease (e.g., Fontaine II or Rutherford 1–3) to ensure relevance to intermittent claudication and generalizability of results, were eligible. Started with SET or ER	Did not meet Markov model criteria
Whyman et al. [20]	Based on duplex imaging	Diffuse disease not identified on duplex but shown on angiogram
Koelemay et al. [21]	Age ≥ 18; Unilateral/bilateral disabling claudication; Walking distance 100–300 m (treadmill test); Iliac/SFA stenosis or occlusion (TASC A–C); Informed consent	Life expectancy < 3 months; Unable to complete forms/consent; Contrast allergy; Pregnancy; Anticoagulant contraindication; Recent symptoms < 3 months; SET history; Heart failure/angina NYHA I/IV; Renal insufficiency (> 150 µmol/L); Participation in another trial

### 2.4 Data Extraction

Data were separately retrieved from the chosen studies by two reviewers. Any disagreements among the reviewers were settled through dialog or, if required, third‐party arbitration. Study information (study design, country, year, and author), participant characteristics (smoking status, sex, age, comorbidities), and intervention details including intervention type, duration, frequency, and delivery mode were extracted from each included study, and outcome measures (MWD, ABPI, QoL, MACE, mortality, complications, reintervention rates) were among the data that were extracted. Additionally, the authors derived data concerning Noninvasive interventions; they are characterized as interventions applied without any endovascular or open surgical procedure. Noninvasive treatments such as SET, a guided walking program under the control of a physiotherapist or clinician, Home‐Based Exercise Therapy (HBET) of a patient‐centered walking regimen, sometimes with the help of a fitness tracker or remote follow‐up. Similarly, BMT, the pharmacological treatment that comprises the use of antiplatelet drugs (aspirin/clopidogrel), statins, antihypertensives, and smoking reduction measures, and lifestyle and risk factor reduction, which includes dietary advising, physical activity recommendations, and nutrition support where reported. When studies presented outcome measures at multiple follow‐up intervals, 2 to 7 years, the longest available between 2 and 7 years, prioritized 2 years when present. This approach ensured comparability across the data set.

### 2.5 Risk of Bias Assessment

To evaluate the risk of bias in the included trials, the Cochrane Risk of Bias Tool (RoB 2) for randomized trials was used. The cluster‐RoB‐2 version was not applied, as no included trials used a clustered randomization design. Some of the domains that were checked for possible bias include random sequence generation, allocation concealment, blinding of participants and of outcome assessors, inadequate outcome data, selective outcome reporting, and others. The interventions are then categorized as first or second tier depending on the evaluations made, which include low risk of bias, some concerns, or high risk of bias.

### 2.6 Data Synthesis and Analysis

Meta‐analyses were performed using Cochrane Review Manager 5 (RevMan 5) when two or more studies reported comparable outcomes. For continuous outcomes (e.g., MWD, ABPI, QoL), mean differences (MD) were calculated when outcomes used the same scale, and standardized mean differences (SMD) were used for differing scales. With continuous data in unequal scales like the Walking Impairment Questionnaire (WIQ) or VascuQoL, appropriate inversion of score was conducted where required. In particular, where worse results indicated a higher value on a scale, scoring was reversed by the formula: inverted score = (maximum possible score + minimum possible score) ‐ original score. This ensured that in all of the experiments, a higher score would always mean a greater number of positive patient values and that the SMDs could be computed meaningfully. Moreover, this operation was consistently applied to all relevant scales before the calculation of SMDs.

For dichotomous outcomes (e.g., MACE, mortality, complications, reintervention rates), odds ratios (OR) were computed with 95% confidence intervals (CI). A random‐effects model was applied due to anticipated heterogeneity, assessed using the I² statistic (I² > 50% indicating significant heterogeneity). To test the robustness of the findings, a sensitivity analysis was conducted excluding studies judged to have a high risk of bias in one or more RoB‐2 domains. This approach helped assess whether any single high‐risk study unduly influenced the pooled estimates.

Similarly, subgroup analyses were planned to investigate sources of heterogeneity (e.g., type of intervention, duration of follow‐up, risk of bias). Nevertheless, these analyses could not be conducted because few studies documented common variables in detail. This restriction did not allow further exploration of the heterogeneity observed. Similarly, measures were reported over different periods of follow‐ups, 2 to 7 years. Data with the longest follow‐up were, where possible, prioritized 2 years when present. Nonetheless, variability in time point and lack of complete reporting in studies did not allow a fully stratified analysis by time. This shortcoming can have an influence on the interpretability of the effect sizes pooled.

### 2.7 Quality of Evidence Assessment

The GRADE approach was used to assess the certainty of evidence for each key outcome (e.g., MWD, ABPI, QoL, MACE, mortality). Judgments were based on study limitations, inconsistency, indirectness, imprecision, and publication bias. A Summary of Findings (SoF) table is provided (See Appendix) in accordance with GRADE guidelines.

## 3 Results

### 3.1 Study Selection

**Figure 1:**
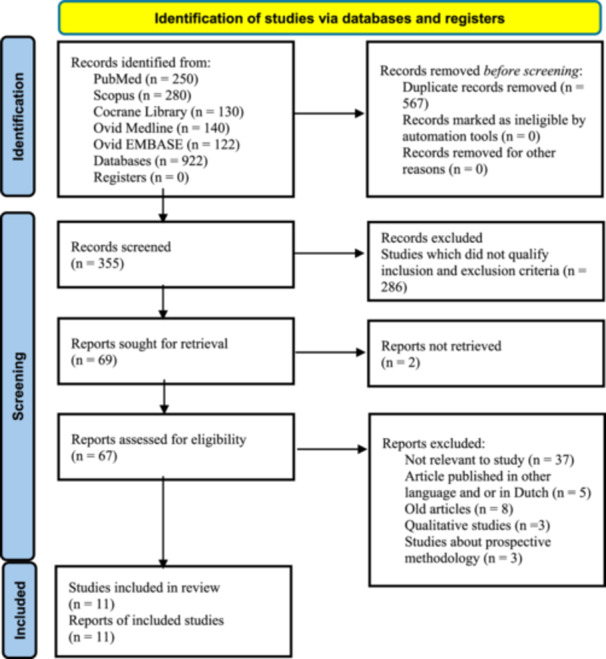
PRISMA chart for included studies.

A total of 922 records were identified through the initial database search. A total of 567 duplicate and irrelevant records were excluded based on their titles. Subsequently, 355 records were screened based on their titles and abstracts. Of these, 286 records were excluded due to irrelevance, leaving 67 full‐text articles assessed for eligibility. Thirty‐seven reports were excluded because they were irrelevant to the study, three were excluded as they were prospective methodologies, and eight were in a non‐English language (Dutch). Ultimately, 11 randomized controlled trials (RCTs) involving 1379 patients were included in the final analysis. The study selection process is summarized in Figure 1.

### 3.2 Study Characteristics

The included studies spanned multiple countries and study designs. The study period ranged from 5 to 7 years, with most studies being multicentre trials. Most of the included trials were conducted in Europe, particularly in the Netherlands and the UK. The patient populations consisted of adults diagnosed with IC who underwent invasive treatments (such as angioplasty or revascularisation) or Noninvasive treatments (including BMT or SET). The individual characteristics of the included studies were summarized in Table 3. In addition to this, the nature and the strength of interventions in the included studies were also different. Percutaneous transluminal angioplasty (PTA) and selective stenting, and open surgical bypass were invasive interventions employed according to the location of the lesion and the type of trial undertaken. SET was usually Noninvasive and varied between 6 and 12 weeks in duration and involved one to three sessions per week, and was performed in hospitals or in outpatient physiotherapy units. The various studies adopted the use of HBET, and these studies involved giving out walking recommendations coupled with calls or the use of pedometers as reminders. The BMT, prescribed in all studies, included antiplatelets (aspirin or clopidogrel), lipid‐lowering drugs (statins), support in quitting smoking, and modifying the risk factor. The characteristics of interventions are described in detail in Table 3. In the studies included, the Noninvasive comparators appeared mostly in the form of SET or HBET along with BMT. None of the studies reviewed focused on unconventional treatment, including thermal intervention, vibrational interventions, or regenerative interventions.

**Table 3:** Individual characteristics of included studies.

Studies	Intervention	Total number of participants	Age		Male Number		Smoking		Comorbidities	
			Control	Invasive	Control	Invasive	Control	Invasive	Control	Invasive
Djerf et al. [12]	MBT vs BMT + revascularization (open surgery OR Angioplasty)	158 Control: 79 Invasive: 79	NR	NR	NR	NR	NR		Same as the IRONIC study above	Same as the IRONIC study above
Fakhry et al. [7]	SET vs ER	151 Control: 75 Invasive: 76	66	65	39	44	17/32	(12/40)	DM: 15 HTN: 28 Hyperlipidemia: 38 IHD: 21 Pulmonary disease: 9 Osteoarthritis: 5 Renal insufficiency: 3 Cardiovascular disease: 4	DM: 11 HTN: 32 Hyperlipidemia: 40 IHD: 14 Pulmonary disease: 7 Osteoarthritis: 7 Renal insufficiency: 1 Cardiovascular disease: 8
Greenhalgh et al. [13]	SET + BMT vs SET + BMT + PTA	93 Control: 45 Invasive: 48	68.5	63.9	26	33	38	38	BMI: 26.9 HTN: 34 IHD: 10 Using statins: 30	BMI: 27 HTN: 35 IHD: 21 Using statins: 40
Gunnarsson et al. [14]	MBT vs BMT + SFA angioplasty	63 Control: 32 Invasive: 31	69.8	71.3	28	22	Current: 11 Previous: 32	Current: 7 Previous: 27	Low density lipoprotein = mmol/L 2.55 ± 0.9 Blood glucose = mmol/L mmol/L 6.3 ± 2.1 Systolic blood pressure = mmHg 150 ± 20.7 Diastolic blood pressure = mmHg 79 ± 8.8 Serum creatinine = mmol/L 82 ± 21.9	Low density lipoprotein = mmol/L 2.75 ± 1.1 Blood glucose = mmol/L 7.0 ± 2.8.21 Systolic blood pressure = mmHg 155 ± 21.7 Diastolic blood pressure = mmHg 80 ± 11 Serum creatinine = mmol/L 84 ± 24.5
Koelemay et al. [21]	SET vs ER for Patients with iliac artery stenosis or occlusion	240 Control: 114 Invasive: 126	63	61	57	31	Current: 60 Former: 48 Never: 6	Current: 68 Former: 53 Never: 5	Hypertension: 54 Hypercholesterolemia: 64 Diabetes: 19 Ischemic heart disease: 23 TIA: 6 Stroke: 4 Mild COPD: 19 Severe COPD: 1 Previous musculoskeletal disorders: 13 Current musculoskeletal disorders: 6	Hypertension: 60 Hypercholesterolemia: 82 Diabetes: 26 Ischemic heart disease: 41 TIA: 8 Stroke: 5 Mild COPD: 25 Severe COPD: 1 Previous musculoskeletal disorders: 7 Current musculoskeletal disorders: 5
Mazari [15]	SEP vs PTA	120 Control: 60 Invasive: 60	75	74.5	NR	NR	9	4	NR	NR
Nordanstig et al. [16]	MBT vs BMT + revascularization (open surgery OR Angioplasty)	158 Control: 79 Invasive: 79	68	68	42	41	Current: 22 Previous: 32	Current: 24 Previous: 32	DM: 16 BMI: 26 Renal failures: 1 IHD: 10 CVA: 9 COPD: 5	DM: 14 BMI: 26 Renal failures: 3 IHD: 12 CVA: 7 COPD: 10
Nylaende et al. [17]	BMT vs BMT + PTA	56 Control: 28 Invasive: 28	69	68	15	16	Current: 19 Previous: 0	Current: 20 Previous: 6	Treated hypercholesterolemia: 6 Untreated hypercholesterolemia: 20 DM: 6 COLD: 2 CHD: 5 BMI: 25 HbA1c % 6.2	Treated hypercholesterolemia: 8 Untreated hypercholesterolemia: 16 DM: 4 COLD: 2 CHD: 2 BMI: 26 HbA1c % 6
Perkins et al. [18]	PTA vs SET	56 Control: 26 Invasive: 30	No significant difference ‐ no data		No significant difference M/F		NR	NR	NR	NR
van den Houten et al. [19]	SEP vs endovascular revascularization	309 Control: 159 Invasive: 150	66	66	NR	NR	Reported significant difference, no data	Reported significant difference, no data	NR	NR
Whyman et al. [20]	PTA vs conventional medical treatment	62 Control: 32 Invasive: 30	62.6	60.6	28	23	16	15	DM: 1 BMI 26.20 Mean systolic blood pressure = 155.4	DM: 4 BMI 25.78 Mean systolic blood pressure = 175.9

Characterization of Invasive and Noninvasive interventions					
Study	Invasive Intervention	Noninvasive Intervention	SET Duration	SET Frequency	Delivery Mode
Djerf et al. [12] (IRONIC)	Angioplasty, stenting, or bypass surgery	SET + BMT (antiplatelets, statins, risk factor control)	12 weeks	2–3 sessions/week	Supervised (outpatient)
Fakhry et al. [7]	Angioplasty ± stenting	SET + BMT (antiplatelets, statins, lifestyle advice)	12 weeks	2 sessions/week	Supervised (hospital rehab)
Greenhalgh et al. [13]	PTA (no stenting)	SET + BMT (antiplatelets, statins, smoking cessation)	6 months	1 session/week	Supervised (clinic‐based)
Gunnarsson et al. [14]	Bare‐metal nitinol SFA stents	Home‐based walking advice + BMT (aspirin/clopidogrel, statins)	Not structured	Advised walking; follow‐up at set points	Home‐based (pedometer‐tracked)
Koelemay et al. [21]	Not applicable	SET: treadmill to pain + gait/endurance training, homework, goal setting	6 months	2×/wk → 1×/wk → 1×/2 wk	Supervised outpatient PT
Mazari et al. [15]	PTA only	SET + BMT (aspirin, statins, lifestyle changes)	12 weeks	3 sessions/week	Supervised
Nordanstig et al. [16]	Endovascular ± stenting or open surgery + BMT	Home‐based walking + BMT	24 months	No fixed frequency	Home‐based
Nylaende et al. [17]	PTA + OMT	OMT only: advice to walk, smoking cessation, aspirin/statins, Mediterranean diet	Not structured	Twice daily walking is advised	Home‐based
Perkins et al. [18]	PTA	Exercise training (unspecified structure)	15 months	Not specified	Unspecified
van den Houte et al. [19]	Endovascular or open revascularization	SET (≥ 2/wk, ≥ 6 wks); HBET (advice + tracker, 1–2 follow‐ups/wk)	≥ 6 weeks	SET: ≥ 2/wk; HBET: 1–2/wk follow‐up	SET: Supervised; HBET: Home
Whyman et al. [20]	PTA (no stenting)	Advice to walk frequently, aspirin, and lifestyle counseling	Not structured	No formal sessions	Home‐based

### 3.3 Clinical Outcomes

#### 3.3.1 Maximum Walking Distance (MWD)

**Figure 2:**
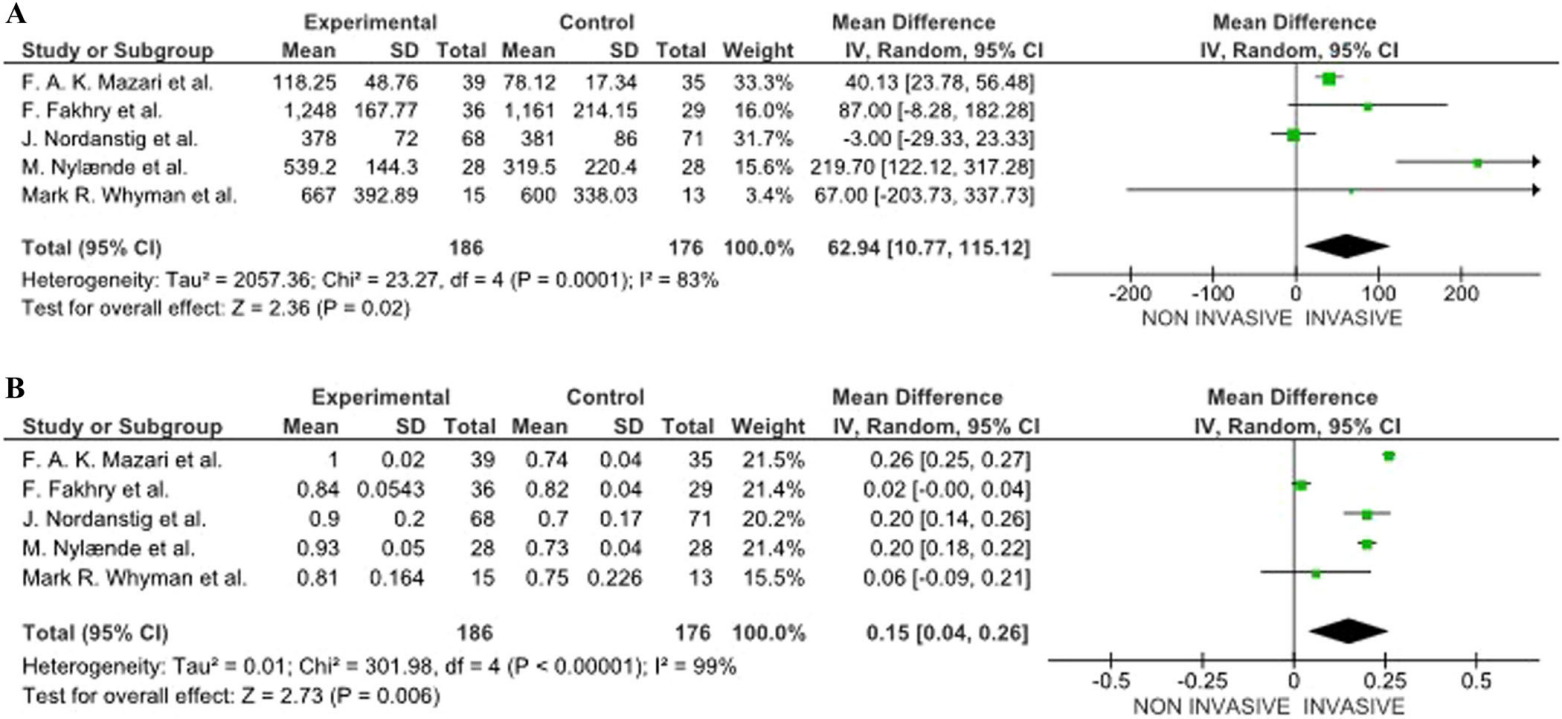
(A) Maximum walking distance (MWD) changes between the groups. (B) Ankle‐Brachial pressure index (ABPI) changes between the groups.

MWD was reported in 5 studies [7, 15, 16, 17, 20], with follow‐up varying from 2 to 7 years. Across all studies, invasive treatments, including percutaneous transluminal angioplasty (PTA), stenting, or open revascularization, significantly improved MWD compared to Noninvasive treatments (SET or BMT). Patients who underwent angioplasty or revascularization demonstrated greater increases in MWD, with an average improvement of 62.94 meter, the overall mean difference, which is positive (62.94) and falls within the confidence interval (pooled MD = 62.94 [10.77, 115.12] 95% CI, *p* = .02, 5 studies), and Z statistic (Z = 2.36) indicate that the overall effect is statistically significant, meaning there is a meaningful difference among the control and experimental. Moreover, the I² statistic (83%) indicates high heterogeneity among the studies, suggesting that the results vary significantly across the included studies. This finding suggests that invasive treatment enhances mobility and greater physical function in patients with IC (Figure 2A). Subgroup or meta‐regression analyses were planned to investigate the source of this heterogeneity but could not be performed due to insufficient data and variability in outcome definitions and reporting across the included trials. This should be considered when interpreting pooled estimates.

Sensitivity analysis excluding high‐risk studies yielded consistent results, with invasive treatments remaining superior to Noninvasive approaches as shown in Figure 2A.

#### 3.3.2 Ankle‐Brachial Pressure Index (ABPI)

The findings suggest that invasive treatments, such as angioplasty or revascularization, lead to a statistically significant improvement in ABPI, which indicates better blood flow in the lower limbs with follow‐up ranging from 2 to 7 years. Specifically, pooled data from five studies showed a mean ABPI increase of 0.15 for patients undergoing invasive procedures compared to those receiving Noninvasive care (SET, or BMT) [7, 15, 16, 17, 20]. Furthermore, data from five of these studies demonstrated a pooled mean difference (pooled MD = 0.15 [0.04, 0.26] 95% CI, *p* = .006, 5 studies), confirming that the improvement is both clinically relevant and statistically significant. This supports the effectiveness of invasive interventions in enhancing peripheral circulation, as shown in Figure 2B. Excluding high‐risk studies did not materially alter the pooled effect estimates, confirming the robustness of the observed improvement in ABPI following invasive treatment (Figure 2B).

#### 3.3.3 Quality of Life (QoL)

**Figure 3:**
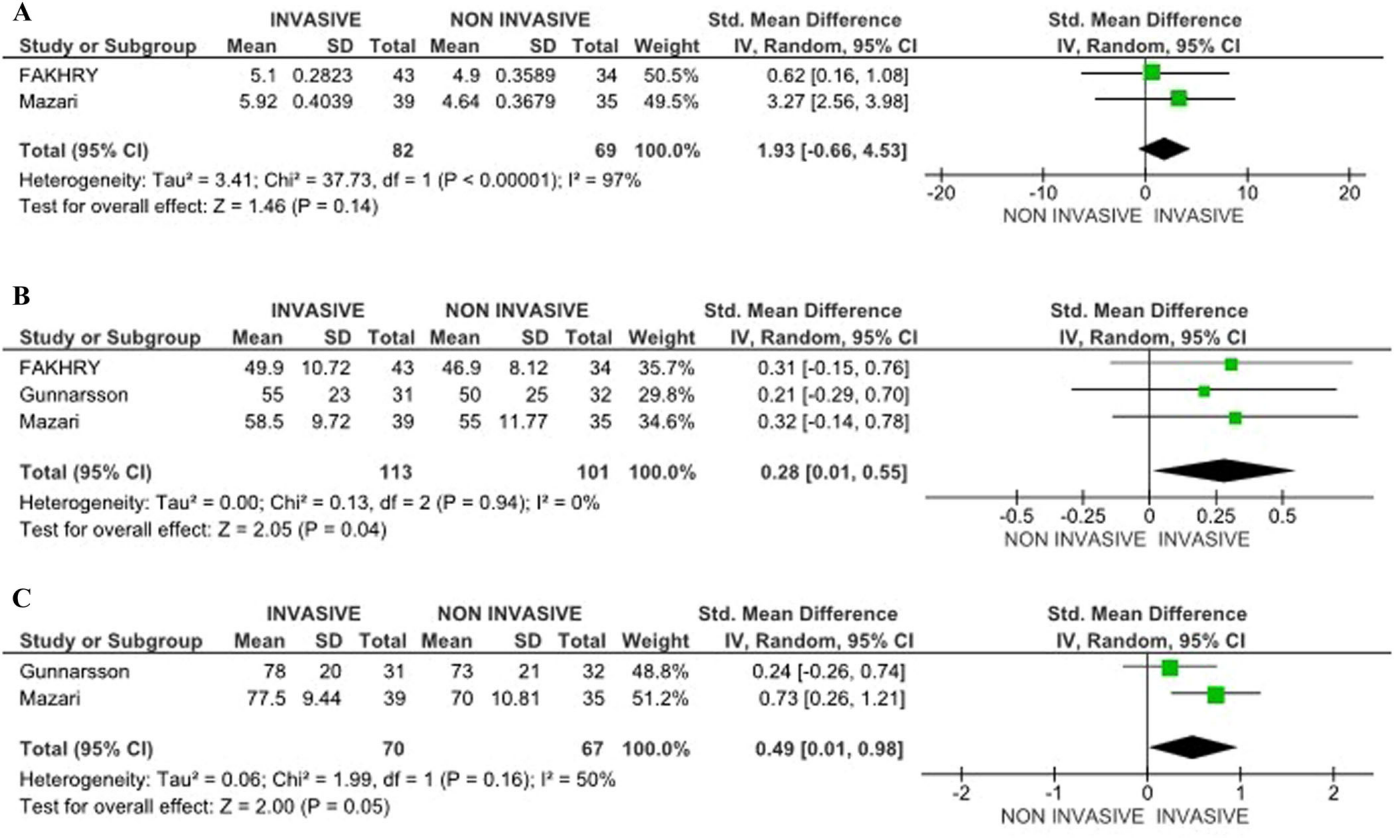
(A) RCTs comparing quality of life (QoL) between the groups using VASCU QoL scales. (B) RCTs comparing quality of life (QoL) between the groups using SF‐36 Physical health outcomes. (C) RCTs comparing quality of life (QoL) between the groups using SF‐36 mental health outcomes.

Results on quality of life were mixed, with follow‐up 2 to 7 years. Some studies showed no significant difference between invasive such as endovascular revascularization (ER), and Noninvasive treatments (SET) when measured using the SF‐36. The VASCU QoL indicated a preference for invasive treatment; however, the results were not statistically significant (Pooled SMD = 1.93 [−0.66, 4.53] 95% CI, *p* = .14, 2 studies) (Figure 3A). Noninvasive treatments, particularly SET, were associated with improvements in specific domains, such as social functioning, which did not differ significantly from invasive treatments [7, 15].

Furthermore, invasive treatments, including the stenting group, were associated with a significant improvement in physical health outcomes with a follow‐up period of 2 to 7 years, including enhanced walking capacity and reduced physical limitations. Relating to the Walking Impairment Questionnaire (WIQ), denoting walking ability, which is a subjective measure, depicted a statistically significant pathway within the stenting group at 36 months and the control group. At 60 months, there were no significant differences in the WIQ scores between the groups, even though the stenting group still had higher scores [14]. Likewise, patients in the invasive treatment group reported fewer difficulties in performing daily physical activities, such as walking up stairs and carrying out household tasks, compared to those in the Noninvasive group (BMT) (Pooled SMD = 0.28 [0.01,0.55] 95% CI, *p* = .04, 3 studies) [7, 14, 15] as indicated in Figure 3B.

The mental health benefits of invasive treatments were less pronounced compared to physical health improvements, with a follow‐up period from 2 to 5 years. However, patients who underwent invasive procedures stenting or PTA, reported a modest reduction in anxiety and depressive symptoms due to the perceived improvement in their physical capabilities and reduced discomfort during physical activities. Noninvasive treatments, particularly SET, were associated with better overall mental health outcomes, as patients engaged in regular exercise experienced mood improvements and enhanced social interactions (pooled SMD = 0.49 [0.01,0.98] 95% CI, *p* = 0.05, 2 studies) (Figure 3C) [14, 15]. Sensitivity analysis restricted to low‐risk studies showed similar trends, with no substantive changes in physical or mental health outcomes, although the precision of estimates was reduced (Figure 3A–C).

#### 3.3.4 Morbidity and Mortality

##### 3.3.4.1 Complications and Major Adverse Cardiovascular Events (MACE)

**Figure 4:**
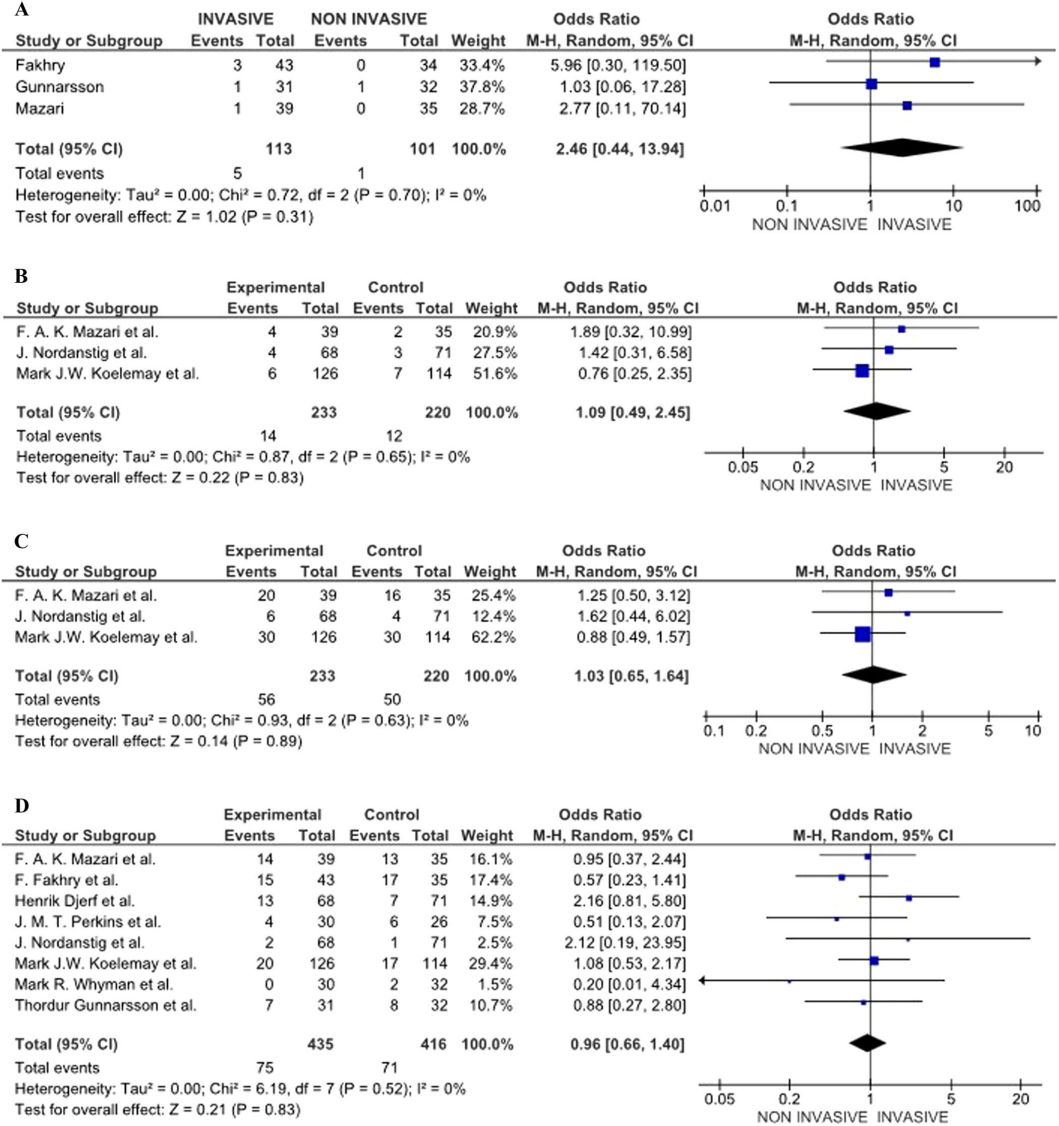
(A) RCTs comparing amputation rates. (B) RCTs comparing Myocardial infarction rates. (C) RCTs comparing major adverse cardiovascular events (MACE) rates. (D) RCTs comparing the mortality rate between the groups.

The rate of complications, including infections and restenosis, was higher in the invasive treatment group, including endovascular and PTA, with a follow‐up period of 2 to 7 years. Although it was not statistically significant, the rate of amputation increased by more than twofold in the intervention group concerning the no‐intervention group (4.4% vs 0.99%) (Pooled OR = 2.46 [0.44, 13.94] 95% CI, *p* = 0.31, 3 studies) (Figure 4A) [7, 14, 15]. Moreover, MACE, including stroke and myocardial infarction, were reported in both groups but were more frequent in the invasive treatment as indicated in 4B (Pooled OR = 1.09 [0.49, 2.45] 95% CI, *p* = .83, 3 studies) [15, 16, 21]. Nevertheless, no significant difference was identified between the two groups in MACE (Pooled OR = 1.03 [0.65, 1.64] 95% CI, *p* = 0.89, 3 studies) (Figure 4C) [15, 16, 21].

##### 3.3.4.2 Mortality

Mortality rates did not differ significantly between the invasive (i.e., PTA or revascularization) and Noninvasive treatment groups (i.e., SET or BMT) with a follow‐up duration between 2 and 7 years. The pooled OR (OR = 0.96 [0.66, 1.40] 95% CI, *p* = 0.83; 8 studies), indicating no statistically significant difference in long‐term survival between the two treatment modalities [7, 12, 14, 15, 16, 18, 20, 21] as indicated in Figure 4D. When high‐risk studies were excluded, the direction and magnitude of effect estimates for complications, MACE, and mortality were unchanged, indicating stable findings (Figure 4A–D).

#### 3.3.5 Re‐Intervention Rates

**Figure 5:**
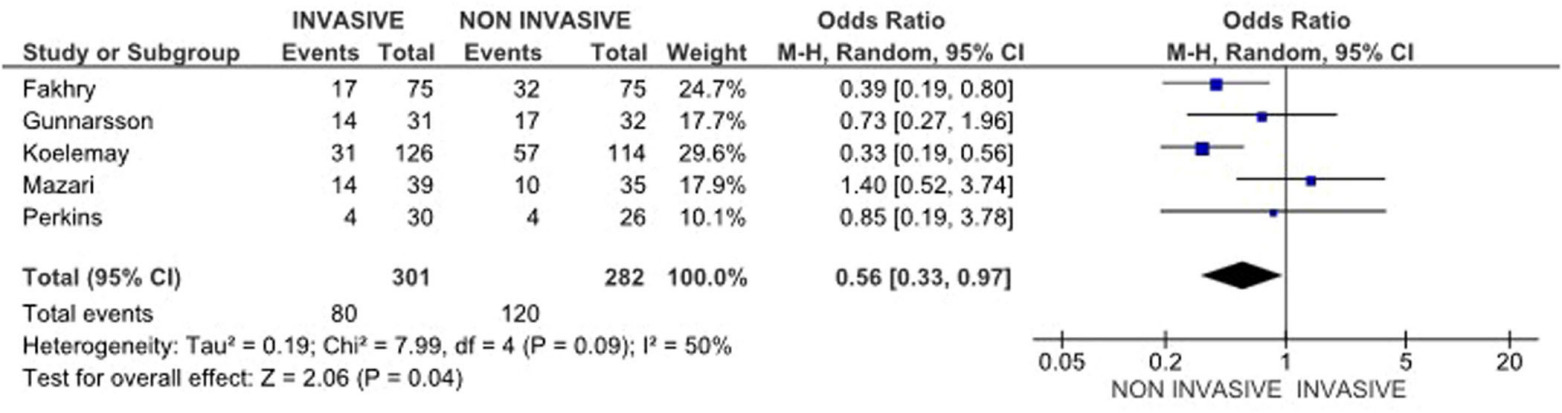
Forest plot of the RCTs comparing re‐intervention rates between the two groups.

Many patients starting with conservative care appropriately escalate to an initial invasive procedure if symptoms persist. These should be described as first invasive procedures rather than re‐interventions, to avoid implying a repeat procedure. Revascularisation patients are at risk of experiencing procedure‐related complications and possible future interventions, and conservatively treated patients (e.g., SET or BMT) can eventually develop into an initial invasive intervention as part of further guideline‐directed therapy. The proportion of patients who experienced conservative management initially and underwent an invasive procedure later in the study (42.6%) was greater in this review than in patients who followed an invasive procedure with a second intervention: usually revascularisation alone without an adjunctive SET or BMT (26.5%) (*p* < 0.01) [7, 14, 15, 18, 21]. Nevertheless, this shift in the conservative group cannot be considered a failure in treatment but is simply standard practice in the clinic, with the invasive procedures being reserved for the patients who fail to respond to the initial Noninvasive medication. Five RCTs with 2 years or more longitudinal follow‐up reported on the subsequent procedures. The cumulative counts of interventions were greater in the conservative compared to the invasive group (Pooled OR = 0.56 [0.33, 0.97] 95% CI, *p* = 0.04) when all procedures were pooled and time‐analyzed. Notably, procedures in the Noninvasive group must not be referred to as re‐interventions, especially physically, in order not to misunderstand the nature of these procedures as the initial ones, as frequently, they are frequently the first invasive interventions after a due Noninvasive intervention. Sensitivity analysis excluding high‐risk studies demonstrated results consistent with the primary analysis, supporting the reliability of the findings (Figure 5).

#### 3.3.6 Cost‐Effectiveness

**Figure 6:**
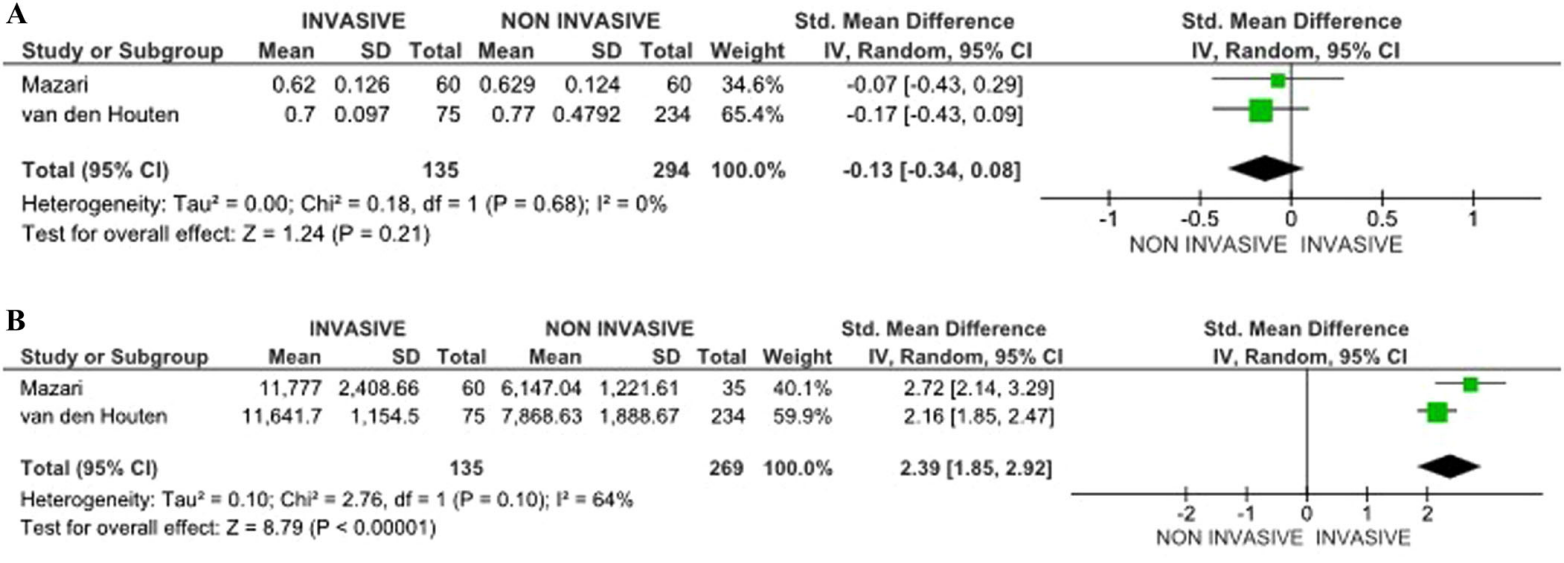
(A) RCTs comparing Cost‐effectiveness analysis (QUALY) (B) RCTs comparing Cost per QUALY between the groups.

**Figure 7:**
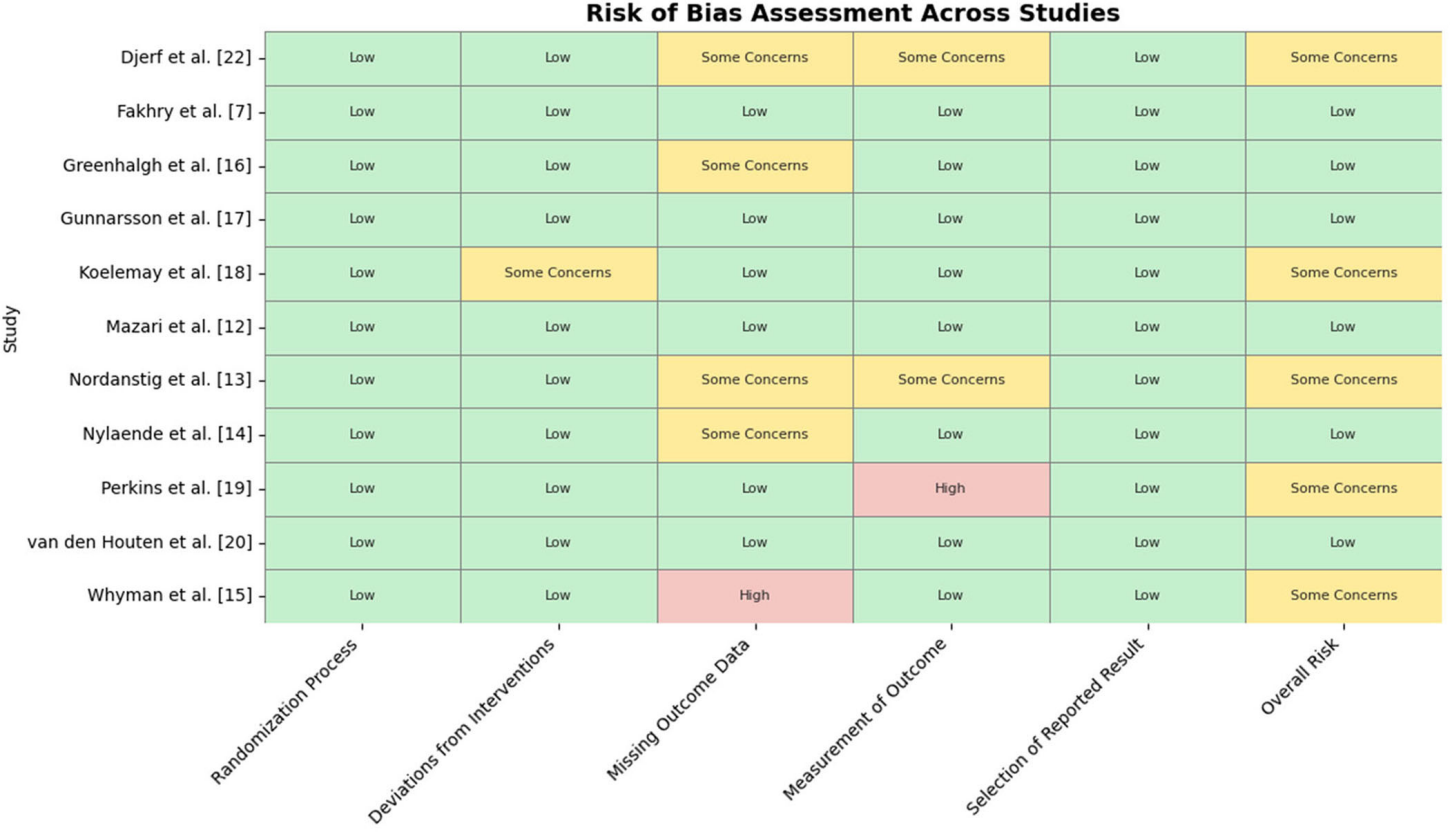
Risk of bias assessment across studies. *Green = Low risk, Yellow = Some concerns, Red = High risk.

The cost‐effectiveness of treatment was examined by adopting the cost per QALY in two studies. They showed significantly higher costs for invasive treatments (PTA or revascularization) compared to Noninvasive treatments (SET or BMT). With follow‐up duration up to 5 years, costs were expressed in 2016 Euros (€), ER was associated with an incremental cost of €91,600 (Euros) per QALY gained in relation to SET in relation to Dutch healthcare payer's perspective. Likewise, SET was associated with cost savings related to ER (−€6412, 95% credibility interval (CrI) –€11 874 to –€1939). The mean difference in effectiveness was −0·07 (95% CrI −0·27 to 0·16) QALY [15, 19]. Moreover, sensitivity analysis was not applicable as only two studies contributed to this outcome, both of which were rated low to moderate risk of bias Figures 6 and 7.

#### 3.3.7 Heterogeneity and Risk of Bias

Heterogeneity was scrutinized using the I² statistic among the included studies. Heterogeneity was observed for MWD (I² = 83%) and ABPI (I² = 99%), while high heterogeneity was noted for QoL outcomes (I² = 97%), likely due to differences in the measurement scales used across studies. Likewise, the RoB 2 tool was used to evaluate the risk of bias. Although several studies raised issues about blinding and insufficient outcome data, most of the research was classified as having a minimal risk of bias. The evaluation of risk of bias was illustrated in Table 4. It presents a summary of the risk of bias across included studies. Most studies showed low risk across domains, although some showed concerns related to outcome measurement or missing data.

**Table 4:** Risk of bias of the included studies.

Study	Randomization process	Deviations from interventions	Missing outcome data	Measurement of outcome	Selection of reported result	Overall risk
Djerf et al. [12]	Low	Low	Some Concerns	Some Concerns	Low	Some Concerns
Fakhry et al. [7]	Low	Low	Low	Low	Low	Low
Greenhalgh et al. [13]	Low	Low	Some Concerns	Low	Low	Low
Gunnarsson et al. [14]	Low	Low	Low	Low	Low	Low
Koelemay et al. [21]	Low	Some Concerns	Low	Low	Low	Some Concerns
Mazari et al. [15]	Low	Low	Low	Low	Low	Low
Nordanstig et al. [16]	Low	Low	Some Concerns	Some Concerns	Low	Some Concerns
Nylaende et al. [17]	Low	Low	Some Concerns	Low	Low	Low
Perkins et al. [18]	Low	Low	Low	High	Low	Some Concerns
van den Houten et al. [19]	Low	Low	Low	Low	Low	Low
Whyman et al. [20]	Low	Low	High	Low	Low	Some Concerns

## 4 Discussion

This systematic review and meta‐analysis suggest that both invasive and Noninvasive treatments effectively manage IC, but with differing strengths and weaknesses. Invasive treatments, such as angioplasty and revascularization, were associated with significant improvements in MWD and ABPI, suggesting better short‐term functional gains compared to Noninvasive treatments such as BMT or SET. However, deciding to opt for invasive treatment must consider the associated risks, including a higher complication rate and careful patient selection [7, 14, 15, 16, 17, 21].

### 4.1 Improved Functional Outcomes With Invasive Treatment

Invasive treatments demonstrated a significant advantage in improving physical function, as evidenced by the increases in MWD and ABPI [7, 14, 15, 16, 17, 21]. The pooled data revealed that patients undergoing invasive procedures, such as revascularization or angioplasty, experienced better improvements in walking capacity and leg blood flow. These outcomes can be attributed to the immediate restoration of arterial patency, which effectively alleviates ischemic symptoms and enhances perfusion. This result is consistent with the results of Rebecca Sorber et al., who reported that early peripheral vascular interventions directed to significant improvements in physical capacity and leg blood pressure among patients with IC [11]. Similarly, Gelin et al. observed sustained enhancements in both leg blood flow and mobility in patients who underwent invasive treatments [10]. These studies collectively underscore the efficacy of invasive strategies in addressing the functional limitations associated with IC, highlighting their role in achieving superior long‐term outcomes compared to conservative approaches.

### 4.2 Risks and Complications of Invasive Treatment

While the benefits of invasive treatment for IC are evident, they are accompanied by an increased risk. The review identified a higher incidence of adverse events, including restenosis, infections, and, in some instances, amputation, although the latter was not statistically significant. Additionally, the MACE occurrence was more frequent among patients who underwent invasive interventions [15, 16, 21]. These findings emphasize the importance of meticulous monitoring and long‐term follow‐up for this patient population. Previous research has also highlighted the potential complications associated with revascularization, particularly in high‐risk patients with comorbid conditions, including hypertension and diabetes. For instance, Golledge et al. reported that patients with IC who experienced early revascularization were at a higher risk of amputation compared to those managed with initial conservative therapy [22]. Similarly, Henrik Djerf et al. emphasized that invasive treatments like angioplasty, stent placement, and bypass surgery carry inherent risks. These include bleeding and hematoma at catheter insertion sites, infections at surgical locations or due to device implantation, and vascular damage, such as arterial dissection or thrombosis [12]. Additionally, the use of contrast agents during imaging was linked to renal complications, and restenosis, a re‐narrowing of treated vessels over time, was a common long‐term concern. Though rare, severe complications occasionally lead to limb amputation.

### 4.3 Noninvasive Treatments and Reintervention

Noninvasive treatments such as SET and BMT were associated with fewer complications but had a higher rate of reinterventions over time. More than 30% of patients treated conservatively required additional procedures within the follow‐up period, compared to only 15% of patients in the invasive treatment group [7, 14, 15, 16, 17, 21]. The observed higher procedural rates among patients initially managed conservatively should not be misinterpreted as a failure of Noninvasive therapy. Rather, they reflect a clinically appropriate escalation of care consistent with established guidelines for peripheral artery disease [23]. The progressive nature of the condition means that a transition to invasive treatment is often anticipated if initial conservative measures prove insufficient. While fewer additional procedures were required among those who began with invasive treatment, this difference highlights treatment sequencing rather than comparative failure rates. Therefore, procedural rates following conservative management should be understood in the context of standard care pathways, not as re‐interventions implying inadequate initial therapy. This suggests that while Noninvasive treatments may delay the need for surgery, they often necessitate reintervention in the long term. These findings highlight the trade‐off between the immediate gains provided by invasive treatments and the potential need for future interventions in conservatively managed patients.

### 4.4 Quality of Life and Patient‐Centered Outcomes

The impact of treatment on QoL varied across studies, with no statistically significant differences observed between invasive and Noninvasive treatments for most QoL measures. However, the general and mental health components of the SF‐36 scale slightly favored invasive treatments, suggesting that patients undergoing revascularization or angioplasty may experience a greater overall improvement in well‐being [7, 15]. This highlights the importance of individualized treatment planning, where both the functional and psychological aspects of the patient's condition are considered. The CLAUDIA trial, a randomized study comparing angioplasty and conservative treatment, found that while angioplasty led to improved walking capacity, the cost‐effectiveness of this invasive treatment was lower compared to SET in the long term. This is while Stewart et al. highlighted that exercise training programs improve walking distance and quality of life in IC patients, even without invasive interventions [24].

These findings highlight the need for a judicious approach when considering invasive treatments for IC. While these procedures offer better functional benefits, clinicians must carefully assess patient‐specific factors and weigh the potential risks against the anticipated gains. This balanced approach is crucial to optimizing outcomes while minimizing the likelihood of adverse events.

### 4.5 Cost‐Effectiveness Considerations

Another critical factor in the treatment decision is cost‐effectiveness. Invasive treatments are typically associated with higher upfront costs due to the need for surgical procedures, hospitalization, and postoperative care. Although this study did not focus extensively on cost‐effectiveness, previous research suggests that the cost per quality‐adjusted life year (QALY) is significantly higher in patients receiving invasive treatments compared to those managed with Noninvasive approaches [7, 15]. However, the long‐term economic impact of reinterventions in conservatively treated patients must also be considered when making clinical decisions.

Several studies utilized tools like the EuroQoL 5‐Dimension 3 L and the Dolan tariff to assess changes in health‐related quality of life (HRQoL) [12, 25, 26]. These instruments evaluate patient health across five domains: depression/anxiety, ability to perform usual activities, discomfort/pain, mobility, and self‐care. By estimating QALY weights at baseline, 12 months, 24 months, and 60 months, they calculated the cumulative QALYs using the area under the curve through linear interpolation between measurement points. Regression analysis was employed to adjust for minor baseline differences in QALY weights between the treatment groups, in line with best practices. These findings emphasize the need for further research to better elucidate the cost–benefit ratio of invasive and Noninvasive treatments, ensuring that clinical and economic factors are comprehensively addressed in treatment planning for IC.

### 4.6 Strengths and Limitations

This review provides a constructive understanding of the long‐term outcomes of both treatment approaches for IC. While invasive treatments (e.g., angioplasty, revascularization) offer better short‐term functional improvements (e.g., walking distance, blood flow), they carry higher risks (complications, MACE) compared to Noninvasive approaches (SET, BMT). Notably, 30% of conservatively managed patients eventually required reintervention, suggesting that Noninvasive therapies delay but do not always prevent invasive procedures. The findings highlight the importance of personalized treatment strategies, weighing immediate functional gains against risks, with careful patient selection critical for optimizing outcomes. A key strength of this analysis is its inclusion of randomized controlled trials, which minimize bias and allow for a high level of evidence. However, several limitations must be acknowledged. First, the heterogeneity observed in the quality‐of‐life outcomes may have affected the overall conclusions, as different studies used various scales to measure QoL. Additionally, the relatively small sample sizes of some included studies and the varying lengths of follow‐up may limit the generalizability of the findings. Furthermore, most of the studies did not prioritize long‐term follow‐up as their primary outcome. Therefore, there is a need for larger, well‐designed trials that are adequately powered to include extended follow‐up periods. Such studies are essential to provide more definitive conclusions on the comparative effectiveness of invasive and Noninvasive treatments.

## 5 Conclusion

In conclusion, both Noninvasive and invasive treatments are effective for managing I, with invasive treatments offering better improvements in ABPI and MWD. However, the higher rate of complications and the associated risks must be carefully weighed when considering invasive options. Noninvasive treatments, although less likely to lead to immediate functional gains, offer a safer approach with fewer complications, but may require re‐intervention in the long term. While our review underscores the need for further high‐quality randomized controlled trials, future research should specifically focus on addressing key gaps by: (1) enrolling more diverse and high‐risk patient populations, including women and older adults; (2) employing standardized, patient‐centered outcomes with long‐term follow‐up; (3) comparing current standard therapies with emerging combination approaches; and (4) integrating cost‐effectiveness analyses to guide health system planning. These targeted improvements will enhance the evidence base and support more personalized, sustainable care pathways for patients with IC. Such studies will provide clinicians with the evidence needed to make informed treatment decisions that balance effectiveness, safety, and quality of life for patients with IC. Moreover, future studies should consider the cost‐effectiveness and patient‐centered outcomes, as highlighted in the PARTNERS program, to ensure that treatment decisions are adapted to the needs and preferences of individual IC patients.

## Author Contributions

**Anas Elmahi:** conceptualization, writing – original draft, methodology, visualization, writing – review and editing. **Nathalie Doolan:** methodology, formal analysis. **Mohiedin Hezima:** methodology, data curation. **Anwar Gowey:** supervision, data curation, methodology. **Daragh Moneley:** formal analysis, methodology, validation, software. **Seamus McHugh:** data curation, formal analysis, software. **Sayed Aly:** methodology, validation, supervision. **Peter Naughton:** supervision. **Elrasheid A. H. Kheirelseid:** investigation, supervision, formal analysis, project administration.

## Conflicts of Interest

The authors declare no conflicts of interest.

## Peer Review

The peer review history for this article is available at https://www.webofscience.com/api/gateway/wos/peer-review/10.1002/cesm.70053.

## Data Availability

No new data were generated, all the data is available within manuscript.

## 1

Table A1

**Table A1:** GRADE‐compliant summary of findings table.

Outcome	Effect	No. of participants (studies)	Certainty of evidence (GRADE)	Comments
MWD	MD = 64.94 meters (95% CI 10.77, 115.12)	5 RCTs, 537 patients	●●●◯ Moderate	High heterogeneity
ABPI	MD = 0.15 (95% CI 0.04, 0.26)	5 RCTs, 476 patients	●●●● High	Consistent findings
QoL	SMD = 0.28 (95% CI 0.01, 0.55)	3 RCTs, 312 patients	●●●◯ Moderate	Scale differences adjusted
MACE	OR = 1.03 (95% CI 0.65, 1.64)	3 RCTs, 379 patients	●●●◯ Moderate	Imprecise estimates
Mortality	OR = 0.96 (95% CI 0.66, 1.40)	8 RCTs, 927 patients	●●●● High	Stable across studies

● = GRADE certainty level

• ●●●● = High

• ●●●◯ = Moderate

• ●●◯◯ = Low

• ●◯◯◯ = Very low
